# Cannabis use in active athletes: Behaviors related to subjective effects

**DOI:** 10.1371/journal.pone.0218998

**Published:** 2019-06-28

**Authors:** Joanna S. Zeiger, William S. Silvers, Edward M. Fleegler, Robert S. Zeiger

**Affiliations:** 1 Canna Research Group, Boulder, CO, United States of America; 2 University of Colorado School of Medicine, Aurora, CO, United States of America; 3 To-Life in Peace, LLC, Wheat Ridge, CO, United States of America; 4 Kaiser Permanente Southern California, San Diego, CA, United States of America; University of Brasilia, BRAZIL

## Abstract

Cannabis use has not been well characterized in athletes. Studies primarily examine problematic use or its categorization by anti-doping bodies as a banned substance. Patterns of use, reasons for use, and responses to cannabis consumption have not been studied in a community-based sample of adult athletes. The Athlete PEACE Survey examined cannabis use patterns and subjective effects to cannabis in a community-based cohort of adult athletes. We used mainly social media and email blasts to recruit and SurveyGizmo to collect data. 1,161 (91.1%) of the 1,274 athletes taking the survey completed it. Current cannabis use was evaluated by asking “In the past two weeks, have you used marijuana (including THC and/or CBD)?” and cannabis type used was assessed by asking “What do you primarily use THC, CBD, or both?”. Cannabis benefits and adverse effects (i.e. subjective effects) and patterns of use were reported. 302 athletes (26%) currently use cannabis of whom 301 had complete data for cluster analysis. Cluster analysis was used to determine cannabis user phenotypes and exploratory factor analysis (EFA) was used to create subjective effects factors. Associations between cannabis user phenotype clusters and the subjective effects factors were explored using multivariate analysis. Cluster analysis identified **t**hree statistically distinct cannabis user phenotypes: (1) older athletes who primarily use medical CBD, (2) mixed age athletes who use cannabis mainly recreationally with both THC and CBD use, and (3) mixed age athletes who used cannabis the longest with primary THC and CBD use. EFA showed three subjective effects factors: (1) Well-being, (2) Calm, and (3) Adverse. Mean positive subjective were higher than mean adverse subjective effects (p<0.001). The cluster using THC and CBD showed the highest mean scores for all three subjective effects factors (p<0.001). Athletes who use a combination of THC and CBD exhibited the most benefit to well-being and calm with minimal adverse effects. Our methodology can be used to develop real-world evidence to inform future use of medical cannabis products.

## Introduction

Athletes often exercise beyond the point of healthy living and develop acute and chronic pain from injuries, overtraining, and too little rest [1,2] The pressure to perform well, particularly during times of injury or performance plateaus, create a situation of increased risk for anxiety, depression, and lack of sleep leading to deficits in well-being [3].

Cannabis has been used in the treatment of pain dating back to 2900 BC [4]. Exogenous cannabinoids are hypothesized to inhibit pain, and a meta-analysis of 28 trials indicated that cannabinoids reduced pain greater than placebo [5] leading to the conclusion that “there is converging evidence to support the notion that marijuana can produce acute pain-inhibitory effects among individuals with chronic pain [5].” However, the efficacy of cannabis-based medicine is inconclusive; a meta-analysis of 24 randomized-controlled clinical trials showed variable improvement in pain scores [6]. This analysis concluded evidence is still limited regarding cannabis-based medicine, but could be effective for neuropathic pain [6].

The International Olympic Committee (IOC) consensus statement on pain in elite athletes concludes with “further research and increased consistency in measures and methods across studies are needed to better understand the incidence and prevalence of analgesic medication use in sport, and the benefits and risks of various pharmacological and non-pharmacological treatments, and their combinations, for specific pain presentations [7].” However, their discussion about pain management in athletes does not include information about cannabis, yet have protocols for use of steroid injections, anticonvulsants, anti-depressants, and opioids [8]. Due to the paucity of research into the efficacy of cannabinoid treatment for pain in athletes the consensus statement concluded “current evidence does not justify the use of cannabinoids for pain management in elite athletes[8].” Research is needed to determine the statement’s validity.

Cannabis use in athletes has been primarily studied in adolescents, elites, and collegiate athletes in an anti-doping or anti-abuse perspective [9–11]. A recent review of cannabis use in elite athletes concluded that there was no evidence for cannabis use as a performance enhancing drug and that cannabis may play a role in pain management and concussion related symptoms [12].

It is still unknown which of the cannabinoids offers the best analgesia leading to cannabis-based formulations of tetrahydrocannabinol (THC), cannabidiol (CBD), or a combination of both THC and CBD (COMBO) in various ratios. THC and COMBO significantly improved neurogenic and chronic pain, muscle spasms, sleep, and appetite [13,14]. THC is considered well-tolerated in most studies; however studies show adverse reactions including: sedation, increased heart rate, dizziness, and nausea [14,15]. CBD is often viewed as a viable alternative to THC due to its non-psychogenic properties and its efficacy against myriad medical conditions [16]. CBD is primarily known for its efficacy against seizure disorders and has also been shown to provide pain relief, anti-spasticity properties [13] and can reduce anxiety [16,17].

Self-reported effects to cannabis use (i.e. subjective effects) are generally described as positive or adverse, and data reduction techniques such as latent class analysis and exploratory factor analysis have been used to more easily interpret long lists of items that cannabis users might endorse [18,19]. Naturalistic settings and questionnaires have been used to measure subjective effects and studies show that subjective effects are stable over time [20]. Subjective effects have been used to predict patterns of use across age ranges [18–21], to examine performance measures after cannabis ingestion [20,22,23], and as an outcome to untangle dose-response effects [24].

We developed The Athlete Pain, Exercise, and Cannabis Experience (PEACE) Survey to examine cannabis use in athletes and its relationship to subjective outcomes of pain and well-being in a large community-based sample of adult athletes. This exploratory analysis applied two data reduction techniques (cluster analysis and exploratory factor analysis) to create cannabis user phenotypes and to determine if these user phenotypes predict positive and adverse subjective effects outcomes. We hypothesized that athletes use cannabis to effectively manage pain and anxiety.

## Methods

### Participants

**Fig 1:**
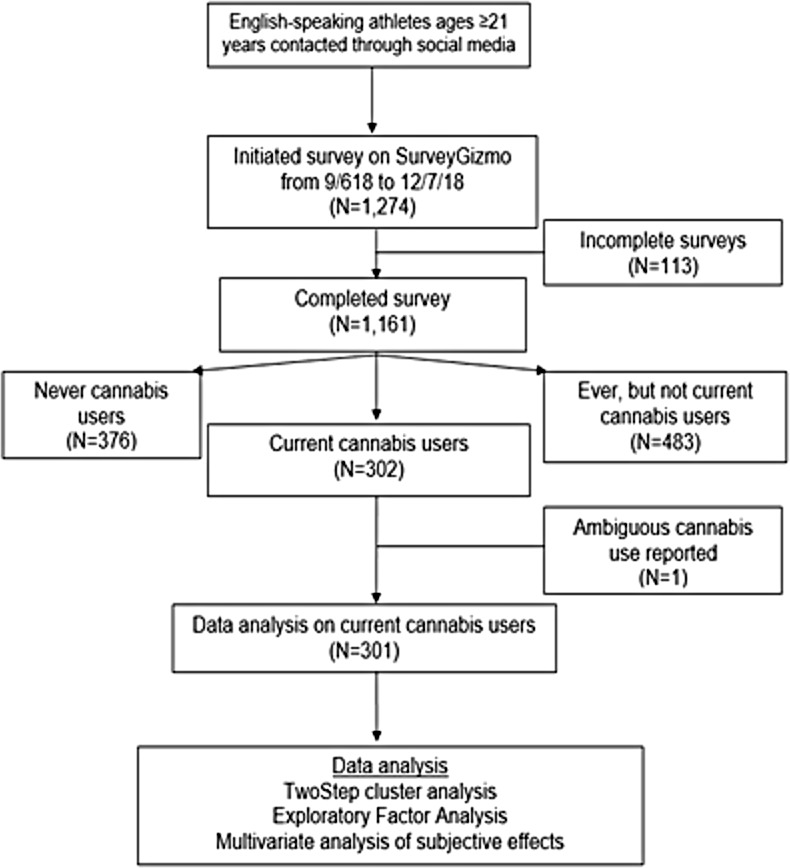
Flow diagram of The Athlete PEACE survey cohort.

This cross-sectional quantitative survey study used a convenience sample. The study was approved with waiver of written consent by Solutions IRB (http://www.solutionsirb.com). Participants were assured confidentiality. Implied consent was provided by survey completion. Participants were required to be, (1) ages 21 years or older, (2) a self-declared athlete of any sport, and (3) English speaking. There were no other inclusions or exclusions. The survey was administered on SurveyGizmo (https://www.surveygizmo.com) between 6 September 2018 and 7 December 2018 (Fig 1) and can be seen in the Supplement (S1 Appendix).

Social media, email communications, and flyers posted in specialty sports stores were used for subject recruitment, allowing for large scale targeting of potential subjects in a relatively short time [25]. Recruitment was researcher-initiated through social media using direct posting of the recruitment call-to-action posted on Facebook pages and dedicated to various endurance athletic sports (e.g. triathlon, swimming, ultra-running, and cycling). Postings were shared by individual athletes on their personal Facebook pages. Postings were also placed on Twitter, LinkedIn, websites dedicated to endurance sports, and email blasts sent directly to coaches and athletes.

### Survey

The development of the questionnaire followed the process used for the development of the athlete mental toughness questionnaire [25]. For creation of the present survey questions we followed the developmental process used in many other cannabis subjective studies published by us [21] and others [20,26]. Subjective effects items were determined by reviewing the literature and amassing the most commonly endorsed items [20,21,26]. The ease of completion and acceptance of the survey is exemplified by the 91% completion rate. Demographics and sports related variables (i.e. primary sport, years in sport, hours per week training, level of athleticism) were collected. Respondents were asked questions about cannabis use which for ease of understanding was generically termed marijuana in the questionnaires. Athletes were asked whether they ever used marijuana and “In the past two weeks, have you used marijuana (including THC and/or CBD)?” Participants who responded “yes” to using marijuana in the past two weeks were asked if they primarily use THC, CBD, or both. Questions about adverse (8 items) and positive (9 items) subjective effects from marijuana use were included. Participants were able to endorse as many of the items that applied to them. Pain was assessed by asking participants whether they have pain (none, <3 months, ≥3 months).

### Analysis

Two data reduction techniques were used to simplify the analyses and to make them more clinically understandable. A TwoStep cluster analysis was used to create cannabis user-types and an exploratory factor analysis was used to create subjective effects scales. All analyses were conducted in IBM SPSS Statistics for Windows, version 24.0 (2016).

Cluster analysis served as an appropriate statistical procedure to divide data into smaller groups with similar characteristics when there are no a priori assumptions about differences within the population; it creates homogenous groups within heterogenous data [27,28]. Cluster analysis was used to identify cannabis user phenotypes to determine how these user phenotypes respond to cannabis, i.e. do subjective effects differ by cannabis user phenotypes? Five cannabis use variables were used in the SPSS TwoStep cluster analysis to create the cannabis user phenotypes. The five included variables were age (21–39 and 40+), frequency of cannabis use (3x weekly or less, 4x weekly to 2x daily, more than 2x daily), duration of cannabis use (less than 3 years, 3 years or more), cannabinoid used CBD, THC, or COMBO), and reason for use (medical, recreational, both). These variables were chosen as each item represents an important motive for use or pattern of use; age was included due to the differences in age-related use behaviors.[29]

Cluster analysis requires, at a minimum, a sample size of 2^K^, where K is the number of variables included in the clustering; it has been suggested that a preferable sample size is 5*2^K^ [30]. Since there were 5 variables in the cluster analysis, a minimum sample size of 32 and a maximum sample of 160 was needed; our sample met these criteria with 301 cannabis-using subjects in the cluster analysis. A systematic analysis of sample sizes for cluster analyses reviewed 243 cluster analyses. The study found that the median sample size for the cluster analyses was 293 participants, similar to the 301 participants used in the present cluster analysis [30]. A simulation study found valid solutions for cluster analysis with samples as small as 20 [31] In addition, the present sample size was adequate to clearly cluster the participants into 3 clinically distinct clusters.

The SPSS TwoStep Cluster method was used to determine both the number of clusters and to allocate subjects to their respective clusters. TwoStep Cluster starts with pre-clustering which uses a sequential clustering approach and then a final clustering using an agglomerative hierarchical clustering method [32]. This is a preferred method of clustering with large datasets where hierarchical clustering can be cumbersome and difficult to interpret and when the number of clusters is not known a priori.

The log-likelihood method with the BIC goodness-of-fit was used whereby a large ratio of distances is considered an optimal number of clusters [32]. Once clusters were identified, post-hoc tests were conducted to determine whether there was inter-cluster heterogeneity and intra-cluster homogeneity. First, the distribution of subjects per cluster was observed (intra-cluster homogeneity) and second, differences for the clustering variables were tested by chi-square to examine cluster separation (inter-cluster heterogeneity).

Subjective effects items were used in a principal components Exploratory Factor Analysis (EFA) with a varimax rotation in an effort to achieve data reduction and data summarization [33]. Sample sizes of more than 100 have been suggested, with a more stringent 20 subjects per item proposed [33]; with 301 subjects endorsing current cannabis use, this study meets those criteria.

Items were retained to a factor if the factor loading was >0.40 and if the item loaded on a single factor (i.e. no cross-loading); factors were retained if they had 3 or more items "to provide minimum coverage of the construct's theoretical domain"[34]. The Kaiser-Meyer-Olkin (KMO) measure of sampling adequacy ranges from 0 to 1 with a value of 0.50 considered adequate and Bartlett’s Test of Sphericity should have a p-values less than 0.05 [35].

The following values of Cronbach’s alpha for establishing the internal consistency reliability were used: Excellent (α>0.9), Good (0.7<α<0.9), Acceptable (0.6<α<0.7), Poor (0.5<α<0.6), Unacceptable (α<0.5) [36,37]; in particular, a value of 0.60 is acceptable for exploratory research and when there are fewer than 10 items in the scale [33].

Summated scales were created for each factor by adding together the items in the factor. This creates the “ability to represent the multiple aspects of a concept in a single measure” and the scales can then be used for multivariate analysis [33].

Multivariate analysis using the SPSS generalized linear model procedure was used to examine whether there were associations between cluster membership and subjective effects. The advantage of multivariate analysis is the ability to assess mean differences across on multiple dependent variables simultaneously with a null hypothesis of equal means across groups [33,38]. A multivariate F-test (Wilks’ Lambda) and partial eta squared (a measure of effect size) were calculated. Estimated marginal means were generated and post-hoc Bonferroni tests were used to examine group differences of these marginal means. P values <0.05, 2 sided, was set for significance.

## Results

### Demographics

Of the 1,274 athlete who started the survey, 91.1% (n = 1,161) completed it (Fig 1). Participants were majority male (62.2%, n = 722), 40 years of age or older (67.8%, n = 787), Caucasian (89.8%, n = 1,042), and participated in three primary sports: triathlon (34.4%, n = 399), running (25.8%, n = 299), and cycling (22.2%, n = 258) with 73.4% exercising ≥5 days/week. Pain was noted in 49.0%(n = 569) (Table 1). 77.1% were athletes for ≥11 years with 46.2% exercising ≥11 hours/week (data not reported).

**Table 1:** Demographics by cannabis use status in 1,161 athletes [Data as N (%)].

Variable	Category	Total (N = 1161)	Current User (N = 302)	Ever, not current User (N = 483)	Never User (N = 376)
Sex^1^	Male	722 (62.2)	182 (60.3)	312 (64.6)	228 (60.6)
	Female	437 (37.6)	120 (39.7)	170 (35.2)	147 (39.1)
Age*	21–39	374 (32.2)	122 (40.4)	139 (28.8)	113 (30.1)
	40 and over	787 (67.8)	180 (59.6)	344 (71.2)	263 (69.9)
Ethnicity	Caucasian	1042 (89.8)	269 (89.1)	439 (90.9)	334 (88.8)
	Other	119 (10.2)	33 (10.9)	44 (9.1)	42 (11.2)
Primary Sport**	Running	299 (25.8)	75 (24.8)	113 (23.4)	111 (29.5)
	Cycling	258 (22.2)	69 (22.8)	111 (23.0)	78 (20.7)
	Triathlon	399 (34.4)	73 (24.2)	184 (38.1)	142 (37.8)
	Other	205 (17.7)	85 (28.1)	75 (15.5)	45 (12.0)
Days per week exercise**	1–4 days	309 (26.6)	112 (37.1)	116 (24.0)	81 (21.5)
	5–7 days	852 (73.4)	190 (62.9)	367 (76.0)	295 (78.5)
Athlete Status*	Professional	25 (2.2)	11 (3.6)	7 (1.4)	7 (1.9)
	Serious/competitive (amateur)	468 (40.3	100 (33.1)	202 (41.8)	166 (44.1)
	Frequent/fitness athlete	405 (34.9)	100 (33.1)	179 (37.1)	126 (33.5)
	Recreational athlete	243 (20.9)	86 (28.5)	87 (18.0)	70 (18.6)
	Other	20 (1.7)	5 (1.7)	8 (1.7)	7 (1.9)
Pain**	No pain	592 (51.0)	118 (39.1)	261 (54.0)	213 (56.6)
	<3 months	94 (8.1)	30 (9.9)	34 (7.0)	30 (8.0)
	3 or more months	475 (40.9)	154 (51.0)	188 (38.9)	133 (35.4)

^1^Not all numbers add to 1,161 due to two participants declining to answer the question. Chi-square test for group differences by cannabis use status

*p<0.01

**p<0.001

### Cannabis use

#### Entire cohort

Ever cannabis use, including current users and ever but not current users, was reported in 67.6% (n = 785) of the athletes. Of the 1,161 participants 302 (26.0%. 95% confidence interval 23.5% to 28.5%) were current cannabis users, 41.6% (n = 483) tried cannabis in the past but were not current users, and 32.4% (n = 376) never used cannabis. (Table 1). 301 of the 302 current cannabis users were included in the remaining analyses due to uninterpretable cannabis data in one participant.

#### Current cannabis users

Of the 301 current cannabis users with complete data, 59.8% (n = 180) were ≥40 years of age and the majority were male (60.1%, n = 181) (Tables 2 and 3), similar to the frequencies of 69.9% ≥40 years of age and 60.8% male observed in the overall participant cohort (Table 1). In current cannabis users, COMBO was the most common cannabinoid type (46.2%, n = 139) and just over half used cannabis ≤3 times weekly and for less than three years (Table 2). Approximately 63% of athletes who used cannabis currently exercised 5–7 days per week with a similar split between the sports and athlete status. More than 50% of athletes who used cannabis currently reported pain lasting more than three months (Tables 3).

**Table 2:** Input variables used to determine cluster membership in 301 cannabis using athletes [Data as N (%)].

Feature	Cluster analysis input variables	Cluster			
		Medical CBD (N = 72)	Mixed Users (N = 152)	Long- duration COMBO (N = 77)	Total (N = 301)
Age**	21–39	14 (19.4)	61 (40.1)	46 (59.7)	121 (40.2)
	40 and over	58 (80.6)	91 (59.9)	31 (40.3)	180 (59.8)
Reason for use**	Medical	72 (100)	25 (16.4)	2 (2.6)	99 (32.9)
	Recreational	0 (0)	72 (47.4)	15 (19.5)	87 (28.9)
	Both medical & recreational	0 (0)	55 (36.2)	60 (77.9)	115 (38.2)
Cannabinoid type**	THC	0 (0)	38 (25.0)	23 (29.9)	61 (20.3)
	CBD	72 (100)	29 (19.1)	0 (0)	101 (33.6)
	COMBO (THC & CBD)	0 (0)	85 (55.9)	54 (70.1)	139 (46.2)
Frequency cannabis use**	3 times weekly or less	33 (45.8)	121 (79.6)	0 (0)	154 (51.2)
	4 times weekly-2 times daily	38 (52.8)	30 (19.7)	49 (63.6)	117 (38.9)
	More than 2 times daily	1 (1.4)	1 (0.7)	28 (36.4)	30 (10)
Duration of use**	< 3 years	71 (98.6)	83 (54.6)	0 (0)	154 (51.2)
	More than 3 years	1 (1.4)	69 (45.4)	77 (100)	147 (48.8)

Chi-square test

^±^p<0.05

*p<0.01

**p<0.001

**Table 3:** Demographic and sports characteristics by cannabis user phenotype cluster [Data as N (%)].

Variable	Category	Cluster			Total (N = 301)
		Medical CBD (N = 72)	Mixed Users (N = 152)	Long-duration COMBO (N = 77)	
Sex	Male	39 (54.2)	92 (60.5)	50 (64.9)	181 (60.1)
	Female	33 (45.8)	60 (39.5)	27 (35.1)	120 (39.9)
Days per week exercise±	1–4 days	21 (29.2)	52 (34.2)	39 (50.6)	112 (37.2)
	5–7 days	51 (70.8)	100 (65.8)	38 (49.4)	189 (62.8)
Primary sport*	Running	14 (19.4)	45 (29.6)	16 (20.8)	75 (24.9)
	Cycling	24 (33.3)	31 (20.4)	14 (18.2)	69 (22.9)
	Triathlon	20 (27.8)	39 (25.7)	13 (16.9)	72 (23.9)
	Other	14 (19.4)	37 (24.3)	34 (44.2)	85 (28.2)
Athlete status*	Professional	2 (2.8)	8 (5.3)	1 (1.3)	11 (3.7)
	Serious/competitive athlete (amateur)	21 (29.2)	50 (32.9)	28 (36.4)	99 (32.9)
	Frequent/fitness athlete	28 (38.9)	52 (34.2)	20 (26.0)	100 (33.2)
	Recreational athlete	21 (29.2)	41 (27.0)	24 (31.2)	86 (28.6)
	Other—Write In	0 (0)	1 (0.7)	4 (5.2)	5 (1.7)
Pain*	No pain	17 (23.6)	69 (45.4)	31 (40.3)	117 (38.9)
	<3 months	5 (6.9)	18 (11.8)	7 (9.1)	30 (10.0)
	3 or more months	50 (69.4)	65 (42.8)	39 (50.6)	154 (51.2)

Chi-square test

^±^p<0.05

*p<0.01

**p<0.001

### Cluster analysis

The SPSS TwoStep procedure automatically selected a three-cluster solution which were named “Medical CBD” (23.9%, n = 72), “Mixed users” (50.5%, n = 152), and “Long-duration COMBO” (25.6%, n = 77) (Table 2). In the Medical CBD cluster participants used only CBD with 100% using cannabis medically. This cluster included 80.6% who were ≥ 40 years of age, 98.6% who used cannabis less than 2 times daily, and 98.6% who used cannabis for less than three years (Table 2). The Mixed users cluster included 59.9% who were ≥ 40 years of age, 99.3% who used cannabis ≤2 times daily with variable reasons for cannabis use, variable types of cannabis used, and variable duration of cannabis use (Table 2). All of the participants in the Long-duration COMBO cluster used cannabis for more than three years with 70.1% using COMBO and 0% using CBD. This cluster skewed younger (59.7%) with 77.9% using cannabis both medically and recreationally and 36.4% using cannabis more than twice daily (Table 2).

All five of the input variables were significantly different between the three clusters. There were no sex differences between clusters. Differences were observed by primary sport, athlete status, days per week of exercise, pain status (Table 3). Athletes in the Medical CBD cluster showed the highest frequency of pain (76.3%) and were mostly cyclists (33.3%). The Long-duration COMBO cluster was characterized by “serious” athletes in the “other” sports category with 60% of these athletes endorsing pain. The Mixed-use cluster had the lowest frequency of pain (55.6%) and they were spread out between the various sports (Table 3).

### EFA of subjective effects

Three items (Euphoria, Gastrointestinal issues, Skin reactions) were removed due to lack of loading on any single factor. A four-factor solution was rejected due to a factor consisting of only two items. A three-factor solution was deemed the best fit with a total explained variance of 47.38 (Table 4). The three factors were named Well-being, Calm, and Adverse. All three factors had an Eigenvalue>1 with Cronbach’s alpha of 0.66 for Well-being and Calm, and 0.65 for Adverse effects. The KMO = 0.78 and Bartlett’s Test of Sphericity was p<0.001. The Calm factor showed the highest relative mean (i.e. a mean of 1.84 from a high score of 3) while the Adverse effects factor showed the lowest relative mean.

**Table 4:** Exploratory factor analysis for positive and adverse effects to marijuana in athletes (n = 301).

Item		Factor Loadings		
	Item Mean	Well-being	Calm	Adverse
Increased energy	0.27	**0.65**	0.31	0.18
Improved athletic performance	0.19	**0.66**	0.26	-0.02
Less pain	0.69	**0.56**	-0.18	-0.02
Fewer muscle spasms	0.17	**0.60**	0.12	0.12
Decreased nausea	0.20	**0.66**	0.25	0.15
Helps with sleep	0.71	0.07	**0.72**	-0.05
Calms me down	0.58	0.20	**0.71**	0.12
Decreased anxiety	0.54	0.30	**0.67**	0.07
Respiratory (e.g. wheezing, coughing, itchy eyes, nasal symptoms)	0.15	0.16	0.08	**0.57**
Cardiovascular (e.g. increased heart rate, palpitations)	0.07	0.14	-0.14	**0.57**
Anxiety, paranoia, feeling uneasy	0.21	0.03	0.01	**0.71**
Difficulty concentrating	0.17	-0.03	0.22	**0.67**
Worse athletic performance	0.03	0.05	0.02	**0.59**
Increased appetite	0.24	-0.04	0.42	**0.47**
**Factor characteristics**				
Cronbach’s alpha		0.66	0.66	0.65
Factor score range		0–5	0–3	0–6
Factor mean (standard deviation)		1.50 (1.35)	1.84 (1.11)	0.65 (1.12)

### Multivariate analysis of subjective effects factors

A multivariate analysis was performed using the three subjective effects factors as dependent variables (well-being, calm, and adverse effects) and cannabis user-type cluster membership as a fixed factor. A statistically significant difference was observed in subjective effects to cannabis based on cannabis user-type cluster membership, (F(9, 721) = 101.61, p<0.001; Wilk’s Λ = 0.14, partial η^2^ = 0.49). Cannabis user-type had a statistically significant effect on Well-being, Calm, and Adverse effects (Table 5).

**Table 5:** Univariate effects in outcome variables.

Outcome variable	df	F	Sig.	Partial Eta Squared
Well-being	2, 298	71.005	<0.001	0.32
Calm	2, 298	51.416	<0.001	0.26
Adverse	2, 298	14.574	<0.001	0.09

**Fig 2:**
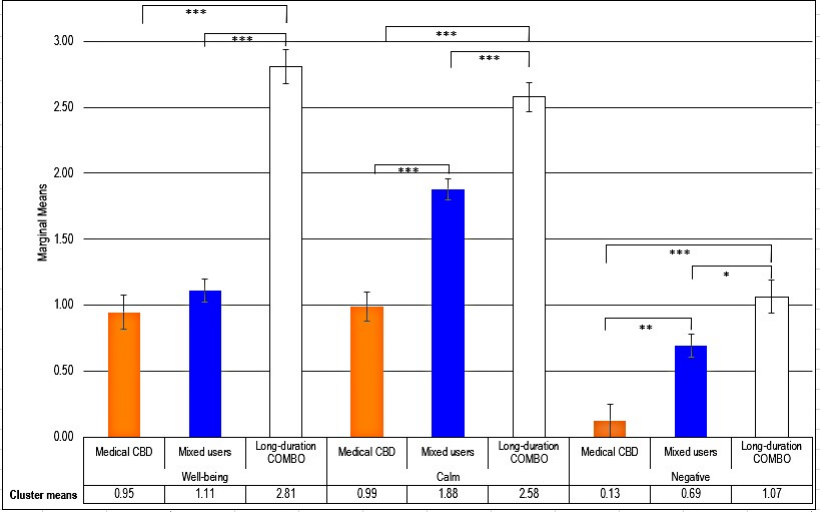
Estimated marginal means from multivariate analysis of subjective effects factors by cannabis user-type clusters. Ranges: Well-being (0–5); Calm (0–3); Adverse effects (0–6); *p<0.05, **p<0.01, ***p<0.001.

The Long-duration COMBO showed the highest means and Medical CBD showed the lowest means for all three subjective effects (Fig 2). Mean scores for Well-being were statistically significantly different between Long-duration COMBO and Mixed users (p< 0.001) and Medical CBD (p<0.001), but not between Mixed users and Medical CBD (p = 1.00). Mean Calm scores were statistically significantly different between Long-duration COMBO and Mixed users (p<0.001), Medical CBD and Long-duration COMBO (p<0.001) and Medical CBD and Mixed users (p<0.001). Finally, mean score for Adverse effects were statistically significantly different between Medical CBD and Mixed users (p<0.01) and Long-duration COMBO (p<0.001), and between Mixed users and Long-duration COMBO (p<0.05).

## Discussion

This cross-sectional survey in 1161 athletes with 301 current cannabis users applied data reduction techniques to examine whether cannabis user-type clusters exist and whether these clusters are associated with subjective effects to cannabis. These analyses revealed (1) three distinct cannabis user-type clusters, (2) differences in subjective effects to cannabis by these cannabis user-type clusters, and (3) combined THC and CBD use provided the largest benefits in well-being and calm. The first cluster (Medical CBD) was characterized by older users who consume CBD for medical reasons. The second cluster (Mixed users) was a mixed-use cluster, meaning that there was variability in the age range and patterns of use. Finally, the last cluster (Long-duration COMBO) used cannabis the longest with an emphasis on COMBO use for medical and recreational reasons. Importantly, positive subjective effects were more often endorsed than adverse subjective effects for all three clusters, and the Long-duration COMBO cluster showed the strongest positive and adverse associations to cannabis use of the three clusters.

Other studies have observed positive benefits from cannabis use. Patients enrolled in the New Mexico Medical Cannabis Program reported “great benefit” from cannabis on quality of life (65%) and activity level (61%) and their reported negative impacts were relatively low (0% for quality of life and 4% for activity level) [39]. Fibromyalgia patients who used cannabis reported strong relief from sleep disorders, improvement in pain and stiffness, they were more relaxed, and reported a higher degree of well-being; at least one adverse side effect was reported by almost all of the participants [40].

Typically, cluster analysis in cannabis research focuses on problem use behaviors [41,42]. Our analyses suggest that adult athletes are using cannabis responsibly and primarily for medical conditions such as pain and anxiety. To this point, of the current cannabis users, less than 30% endorsed recreational-only cannabis use, 10% used cannabis more than twice daily, and 61% indicated they used cannabis for pain. In addition, current cannabis users exercised at a high frequency with about 63% exercising 5–7 days per week and 71% exercising 6–15 hours per week (data not shown), numbers that far exceed the weekly recommendation of 150 minutes per week of exercise [43].

These are impressive physical activity numbers in current cannabis user who reported a pain frequency of 61%. Chronic pain sufferers tend to show lower levels of physical activity than healthy individuals even though there is evidence to support the use of physical activity as a mode of treatment to improve overall health and pain symptoms [44–47]. Early studies suggested that older adult cannabis users engaged in more physical activity (over and above the laboratory exercise provided by the researchers) than non-cannabis users with the hypothesized process being reduced experience of pain in the users [48]. A possible mechanism involves the endocannabinoid system and that this biological system contributes to the cognitive and physiological effects associated with voluntary physical exercise contributing to exercise-induced euphoria [49]. Furthermore, it is hypothesized that there is an exercise-endocannabinoid interaction [50].

The frequency of current cannabis use in the present study is in the higher range noted in a systematic review by Brisola et al. that found the prevalence of current marijuana use among athletes ranged from 10%-24%, with most of these studies conducted in younger adolescent athletes [11]. The frequency of 32.9% medical-only cannabis use was higher in this cohort than the 17% reported in the National Survey on Drug Use and Health[51]. Cross-use of medical and recreational cannabis was observed in 38.2% of our cohort, which is lower than the 55% of medical and recreational combination use seen in a survey of 348 medical cannabis users [52]. Our findings suggest that adult athletes are using cannabis differently than adolescent and university athletes as well as the general population.

Adverse effects were reported at a low frequency amongst the current cannabis users with in the most adverse effects noted in the Long duration COMBO cluster. It is also important to note that studies of initial subjective effects to marijuana (i.e. the effects when using marijuana for the first time) also show a pattern of higher positive responses than adverse responses [21,26].

Athletes exhibit considerable heterogeneity in their age, physical and mental health, athletic ability, and sport of choice. Our survey showed that there is also heterogeneity in cannabis use behavior. Using cluster analysis, we were able to reduce the heterogeneity in cannabis use behavior by identifying sub-groups with similar characteristics and then relating these sub-groups to important outcomes of well-being, calm, and adverse effects. The identified clusters can help athletes and medical practitioners create targeted treatment plans using cannabis. Our results indicate that older athletes who are newer to cannabis use tend to use CBD only; however, in these analyses, CBD alone provided the least reported benefit (albeit, with the least adverse effects). Our analysis cannot capture whether athletes in the Medical CBD cluster move over time to the Long-duration COMBO or Mixed-use cluster as they become more experienced with cannabis use or their symptoms are not helped by CBD alone. It is reassuring that 55% of this cohort reported no adverse effects.

Our results do shed some light on one of the prevailing questions regarding cannabis use for medicinal purposes: which cannabinoid offers the best symptom relief with the fewest adverse effects? CBD has been used for its anti-inflammatory properties and lack of psychogenic effects [13,16]. However, our study suggests that CBD used in combination with THC provides greater analgesic and anti-anxiolytic relief than CBD alone. The question of what ratio to use is still largely unknown, and our questionnaire did not ask about ratios of CBD and THC among those who use both. A recent commentary provided some guidelines: (1) start with CBD extract of 5–10 mg twice daily, (2) increase the dose over 1–2 months until pain relief is achieved, (3) if CBD alone isn’t sufficient for relief, add 1–2.5 mg THC and slowly titrate up as needed [53]. The authors further stated that “CBD also widens THC’s therapeutic window when administered concomitantly, increasing the maximum tolerated dose and decreasing the risk for adverse events”[53].

A common limitation of questionnaire-based studies is recall bias. The ReLeaf App was designed to collect real time self-reported cannabis dosing, reasons for use, and side-effects (positive and adverse). An analysis of ReLeaf App users found that the common reasons for cannabis use were depression, anxiety, and pain with more relief observed for anxiety and depressive symptoms than pain [54]. Our cohort showed higher scores in the Calm scale, which included anxiety, than the Well-being scale which included pain, indicating that our questionnaire adequately captured real-time cannabis use. In ReLeaf App users, higher pre-dosing levels were associated with more symptom relief [54]. We did not collect information on dosing; however, we did find that the cluster with more frequent use showed the highest mean scores for the Well-being and Calm subjective effects factors. ReLeaf App users reported a higher degree of adverse side effects (about 60%) than our cohort, however, the positive side effects were reported more often, at a rate of about 94% [54].

It is not known whether the participants were answering the questions honestly; however, the anonymity of the questionnaire increased the likelihood of truthful responses. The internal consistency of the responses also lends credibility to the participants answers. The generalizability of this convenience sample drawn from social media outlets is unknown. However, comparisons to the latest statistics from the governing body of triathlon (USA Triathlon) and cycling (USA Cycling) show that the participant demographics in this sample roughly match the overall populations, however our runners skew male which is different than the sex composition found by Running USA [55–57]. Even though the sample demographics roughly reflect those of the greater population of triathletes, runners, and cyclists, the participants are self-selected, therefore the cannabis user-type clusters and subjective effects to cannabis may not be representative of athletes in general.

The role of cannabis-based medicine is complex. Meta-analyses have shown limited [58] or no effect for cannabis in treating pain [59]. However, pain is not the only indication for medical cannabis use; studies have shown that cannabis use improved sleep, reduced spasticity, and enhanced health-related quality of life [60–63] benefits which were observed in the current analysis. One way to help unravel the complexities of cannabis-based medicine is cluster analysis. Cluster analysis has been used clinically to create phenotypes to improve clinical practice and treatment recommendations [64,65]. Heterogeneous populations create challenges for clinicians and identifying subgroups can aid in diagnostic criteria and help explain outcomes; “cluster analysis also has the potential to improve our understanding of differential treatment responses in different patient subgroups and to provide more personalized treatment to enhance recovery [of disease].”[64] The present analysis used cluster analysis to successfully create cannabis use phenotypes which were used to help determine who responds to positive and adverse cannabis subjective effects. This is particularly important for medical patients who are seeking relief from a variety of disorders. The cluster phenotypes inform of the patterns of use that might provide such relief.

In summary, the present novel cluster analysis among current cannabis-using athletes found three distinct cannabis user-type clusters that were associated with positive and adverse subjective effects to cannabis. Combination use of THC and CBD offered the most benefit to well-being and calm with low frequency of adverse effects while CBD alone offered the least positive effects. These results add to the conversation concerning how cannabis is used in the real-world to optimize well-being and calm while minimizing adverse effects. Because of the tremendous interest surrounding medical cannabis, other populations can be studied in a similar manner. Insurers, academia, and government might be interested in using our methodology to develop real-world evidence to inform future use of medical cannabis products.

## Supporting information

S1 Appendix The Athlete PEACE survey questionnaire. (DOCX)

## Abbreviations

EFA: exploratory factor analysis
CBD: cannabidiol
THC: tetrahydrocannabinol
COMBO: combination use of THC and CBD
