# Supplementary material for: Cannabis use in active athletes: Behaviors related to subjective effects
Source: PLoS One. 2019 Jun 28;14(6):e0218998. doi: 10.1371/journal.pone.0218998 (PMC6598761; doi:10.1371/journal.pone.0218998)
Supplement: S1 Appendix — (DOCX) [file pone.0218998.s001.docx]

# The Athlete PEACE Survey

## Demographics

#### What is your age?

( ) 21 to 29

( ) 30 to 39

( ) 40 to 49

( ) 50 to 59

( ) 60 to 69

( ) 70 or older

#### What is your gender?

( ) Male

( ) Female

( ) Other - Write In: _________________________________________________

( ) Prefer not to answer

#### What is your ethnicity?

( ) American Indian/Alaska Native

( ) Asian

( ) Black/African-American

( ) Hispanic/Latino

( ) Native Hawaiian or Other Pacific Islander

( ) White

( ) Prefer not to answer

( ) Other - Write In: _________________________________________________

#### What is your employment status?

( ) Full-time

( ) Part-time

( ) Unemployed

( ) Retired

( ) Student

( ) Other - Write In: _________________________________________________

#### What is your highest level of education?

( ) Less than high school

( ) Graduated high school

( ) Trade/technical school

( ) Some college, no degree

( ) Associate degree

( ) Bachelor's degree

( ) Advanced degree (Master's, Ph.D., M.D.)

#### Do you have pain?

( ) I have had pain for 3 or more months

( ) I have had pain for less than 3 months

( ) I don't have pain

## Athlete Profile

#### How many years have you been an athlete?

( ) 1-2 years

( ) 3-5 years

( ) 6-10 years

( ) 11-19 years

( ) 20 years or more

#### What is your primary sport/exercise?

( ) Swimming

( ) Running

( ) Cycling

( ) Triathlon

( ) Yoga/Pilates

( ) Climbing

( ) Spartan races

( ) Trail running

( ) Hiking

( ) Walking

( ) Strength training/gym

( ) Winter sports (skiing, snowboarding, snow shoeing)

( ) Martial arts/MMA

( ) Other - Write In: _________________________________________________

#### Over the past three months, on average, how many ****days per week**** do you exercise (include all sports that you participate in)?

( ) 1

( ) 2

( ) 3

( ) 4

( ) 5

( ) 6

( ) 7

#### Over the past three months, on average, how many ****hours per week**** do you exercise (include all sports that you participate in)?

( ) 0-5 hours

( ) 6-10 hours

( ) 11-15 hours

( ) 16-20 hours

( ) more than 20 hours

#### What is your athletic status?

( ) Professional

( ) Serious/competitive athlete (amateur)

( ) Frequent/fitness athlete

( ) Recreational athlete

( ) Other - Write In: _________________________________________________

## Marijuana Use

#### In the past two weeks, have you used marijuana (including THC and/or CBD)?

( ) Yes

( ) No

#### 36) Why do you currently NOT use marijuana? (check all that apply)

[ ] I don't need to use marijuana

[ ] I don't know enough about marijuana

[ ] Marijuana is not legal in my state

[ ] Marijuana is not legal at my job

[ ] I am scared to use marijuana

[ ] I am allergic to marijuana

[ ] I get sick from marijuana

[ ] I consider using marijuana doping

[ ] I am uncomfortable going to a dispensary

[ ] I can't afford it

[ ] Other - Write In: _________________________________________________

**Do you use marijuana medically, recreationally, or both?**

( ) Medically

( ) Recreationally

( ) Both medically & recreationally

**What do you primarily use THC, CBD, or both?**

( ) THC

( ) CBD

( ) Both THC & CBD

( ) Other - Write In: _________________________________________________

**How long have you been using marijuana?**

( ) 3 to <6 month

( ) 6 months to < 12 months

( ) 12 months to

( ) 3 years or more

( ) Not applicable

( ) Other - Write In: _________________________________________________

#### How often do you use marijuana?

( ) 1-3 times weekly

( ) 4-7 times weekly

( ) 1-2 times per day

( ) More than 2 times per day

( ) Other - Write In: _________________________________________________

( ) Not applicable

#### How do you use marijuana? (check all that apply)

[ ] Smoke in cigarette or pipe

[ ] Vaporizer

[ ] Spray

[ ] Capsule

[ ] Oil/tincture

[ ] Edible (cookie, candy, etc.)

[ ] Topical (cream, ointment, patch)

[ ] Other - Write In: _________________________________________________

[ ] Not applicable

#### 42)

#### Have you had any negative effects from marijuana use or contact? (check all that apply)

[ ] Respiratory (e.g. wheezing, coughing, itchy eyes, nasal symptoms)

[ ] Cardiovascular (e.g. increased heart rate, palpitations)

[ ] Gastrointestinal (e.g. nausea, vomiting, diarrhea)

[ ] Skin reaction (e.g. hives, rash)

[ ] Anxiety, paranoia, feeling uneasy

[ ] Worse athletic performance

[ ] Difficulty concentrating

[ ] Increased appetite

[ ] Other - Write In: _________________________________________________

[ ] No negative effects

#### Have you had positive effects from marijuana use? (check all that apply)

#### [ ] Increased energy

[ ] Helps with sleep

[ ] Euphoria

[ ] Calms me down

[ ] Decreased anxiety

[ ] Improved athletic performance

[ ] Less pain

[ ] Fewer muscle spasms

[ ] Decreased nausea

[ ] Other - Write In: _________________________________________________

[ ] No positive effects

**Do you take marijuana for pain?**

( ) Yes

( ) No
